# Molecular events leading to HPV-induced high grade neoplasia

**DOI:** 10.1016/j.pvr.2016.04.003

**Published:** 2016-04-12

**Authors:** Saskia M. Wilting, Renske D.M. Steenbergen

**Affiliations:** Department of Pathology, VU University Medical Center Amsterdam, The Netherlands

## Abstract

Cervical cancer is initiated by high-risk types of the human papillomavirus (hrHPV) and develops *via* precursor stages, called cervical intraepithelial neoplasia (CIN). High-grade CIN lesions are considered true precancerous lesions when the viral oncogenes E6 and E7 are aberrantly expressed in the dividing cells. This results in abolishment of normal cell cycle control *via* p53 and pRb degradation. However, it has become clear that these viral oncogenes possess additional oncogenic properties, including interference with the DNA methylation machinery and mitotic checkpoints. Identification of the resulting molecular events leading to high-grade neoplasia will 1) increase our understanding of cervical carcinogenesis, 2) yield biomarkers for early diagnosis, and 3) identify therapeutic targets for HPV-induced (pre) cancerous lesions.

This review will briefly summarise current advances in our understanding of the molecular alterations in the host cell genome that occur during HPV-induced carcinogenesis.

## 1 Cervical carcinogenesis and hrHPV-induced transformation

Cervical carcinomas are caused by infection with hrHPV types and can be divided in two main histotypes, squamous cell carcinomas (SCCs; 80%) and adenocarcinomas (AdCAs; 15–20%) [1]. SCCs develop *via* well recognised precursor lesions, called cervical intraepithelial neoplasia (CIN), that according to severity are graded from 1 (mild dysplasia) to 3 (severe dysplasia). Together CIN2 and CIN3 lesions are referred to as high-grade CIN lesions (hgCIN). Little is known about AdCA precursor lesions up to adenocarcinoma-in-situ (ACIS). Progression of hrHPV-infected epithelial cells to invasive cancer is a long term process associated with the accumulation of DNA alterations in host cell genes. These alterations involve both epigenetic and genetic changes in oncogenes and tumour suppressor genes. Epigenetic changes affect gene expression by mechanisms other than changes in the underlying DNA sequence, whereas genetic changes are considered adaptations in the DNA sequence itself.

The process of HPV-mediated transformation and its associated accumulation of crucial (epi)genetic events over time can be mimicked *in vitro* by long term passaging of primary keratinocytes infected with hrHPV. Thereby these *in vitro* models enable longitudinal and functional analysis of driver events.

## 2 DNA methylation

One of the best studied epigenetic mechanisms is DNA methylation, the covalent addition of a methyl group (-CH3) to cytosines preceding guanines in the DNA sequence, called CpG dinucleotides. CpGs can be clustered in so-called CpG islands, which are CpG-rich sequences frequently located in gene promoters. DNA methyltransferases are the enzymes responsible for DNA methylation. De novo DNA methyltransferases DNMT3A and B are involved in the establishment of novel DNA methylation patterns, whereas DNMT1 ensures proper maintenance and inheritance of already established methylation patterns. During cancer development local hypermethylation of the CpG islands located in gene promoter regions can lead to silencing of tumour suppressor genes (reviewed in [2]).

Interestingly, hrHPV E6 and E7 have been found to directly associate with and increase the activity of DNMTs [3], [4], [5]. Silencing of E6 and E7 decreased methylation of tumour suppressor genes and reversed the transformed phenotype of cervical cancer cells [6]. The direct association between the enzymes responsible for DNA methylation and HPV may (partly) explain why DNA hypermethylation is found to be a frequent event during cervical carcinogenesis. In support of this HPV-positive head and neck squamous cell carcinomas (HNSCC) showed higher overall methylation rates compared to HPV-negative HNSCCs [7]. Together these observations suggest that induction of DNA methylation mediated silencing of tumour suppressor genes represents a separate oncogenic property of HPV.

Methylation-mediated silencing of many protein-coding tumour suppressor genes has been described in cervical (pre)cancer (reviewed in [8], [9]). Besides protein coding genes also methylation mediated silencing of non-coding microRNAs (miRNAs) has been detected in cervical lesions (reviewed in [10]). The level of methylation was found to increase with severity of cervical disease and within hgCIN lesions also with the duration of disease [11], [12]. The functional relevance of these methylation events was demonstrated for part of these genes and miRNAs, including C3ORF14, C13ORF18, CADM1, MAL, PRDM14, SFRP2, miR-124, miR-203, and miR-375, using *in vitro* models [13], [14], [15], [16], [17], [18], [19], [20], [21], [22]. Moreover, *in vitro* studies have shown that the onset of DNA methylation may differ between genes, but that in all instances the methylation levels increase during transformation [23], [24]. Interestingly, the methylation patterns were found to be mostly independent of the hrHPV-type present [24]. A recent study in our group highlighted the importance of methylation-mediated silencing of certain miRNAs for the acquisition of anchorage independence during HPV-induced transformation (unpublished data).

## 3 Chromosomal alterations

When aberrantly expressed in dividing cells, e.g. due to viral integration or methylation, both viral oncoproteins E6 and E7 are known to induce DNA damage, centrosome abnormalities and chromosomal segregation defects, thereby leading to chromosomal instability (reviewed in [25], [26]). Next to expression of viral oncogenes, viral integration has been related to chromosomal instability. Two recent independent studies describe that viral integration occurs close to or within amplified chromosomal regions more often than expected by chance [27], [28]. However, it remains a matter of debate whether viral integration results in chromosomal instability or whether chromosomal instability facilitates viral integration. The facts that the frequency of viral integration increases with progression to cancer and that chromosomal instability is also observed in cases without viral integration support the latter [27], [29], [30].

In 2014, Thomas et al. performed a meta-analysis using data from studies that determined chromosomal changes by comparative genomic hybridisation (CGH) in HPV-positive and -negative cancers and in premalignant lesions of the anogenital tract (cervix, anus, vagina, penis and vulva) [31]. In cervical SCC a gain of 3q was the most frequently observed alteration in 55% of cases. Other common alterations include loss of chromosome 3p and 11q, observed in 36% and 33% of SCC. These findings are corroborated by recent exome-sequencing studies [28], [32]. Gain of 3q and loss of 3p and 11q were less common in cervical AdCA, which instead more frequently showed a gain at 17q (36%).

The three studies on hgCIN lesions that were available for this meta-analysis showed that the most common alterations in SCC were already observed in part of the hgCIN lesions (gain of 3q in 27%, loss of 3p and 11q in 8% and 15% respectively), suggesting these alterations to be crucial and their presence is likely to reflect advanced hgCIN. Interestingly, when comparing chromosomal profiles of histologically similar hgCIN lesions great heterogeneity was observed [33]. Whereas part of the CIN lesions showed little to no alterations, others had chromosomal profiles comparable to invasive carcinomas. HgCIN lesions with these ‘cancer-like’ profiles were found to most likely represent longer lasting, more advanced lesions as indicated by the duration of preceding hrHPV infection [34].

Although sample sizes of most individual studies did not allow for reliable comparison between chromosomal profiles of lesions with different HPV-types, Thomas et al. were able to investigate this in their meta-analysis [31]. Gain of 3q was significantly more common in HPV16-induced squamous lesions, whereas gain of 17q was more common in HPV18-induced AdCA compared to other HPV types. This may reflect HPV type dependence, as supported by recent *in vitro* work of our own group. It was found that the number of observed chromosomal aberrations in HPV-immortalised keratinocytes is HPV-type dependent and inversely related to the viral immortalisation capacity [35].

## 4 Mutations

The role of somatic mutations during HPV-induced carcinogenesis has long remained inconclusive. Recent profiling of 301 cervical carcinomas using a mass spectrometry-based panel of known somatic mutations (GynCarta 2.0, Sequenom) revealed frequent mutations in PIK3CA (SCC 25%, AdCA 3%) and KRAS (SCC 11%, AdCA 24%) [36]. A comprehensive genomic landscape paper on 115 cervical carcinomas revealed both known and novel recurrent mutations in cervical SCC, including mutations in EP300 (16%), FBXW7 (15%), PIK3CA (14%), HLA-B (9%), p53 (9%), MAPK1 (8%), PTEN (6%), ERBB2 (5%), STK11 (4%), NFE2L2 (4%) [28]. With the exception of PIK3CA (16%), cervical AdCA harboured recurrent mutations in another subset of genes including ELF3 (13%), KRAS (8%), and CBFB (8%). In addition, differences in mutational signatures were observed between SCC and AdCA. Whereas SCC predominantly showed a Tp^*^C mutational signature, AdCA mainly contained *CpG signatures. Interestingly, Tp*C mutations have been associated with the APOBEC family of enzymes, known to be involved in the cellular response against HPV [37].

To date, little data exists on somatic mutations in hgCIN lesions. By mutation analysis of 48 cancer-related genes using a targeted sequencing approach in a panel of SCCs, AdCAs and hgCIN lesions, PIK3CA was the only gene found to be mutated in a single hgCIN case [38]. Subsequent high-resolution melting analysis of 209 hgCIN lesions, revealed PIK3CA exon 9 hotspot mutations (p.E542K and p.E545K) at very low levels in only 2.4% of these lesions, suggesting that mutations of PIK3CA represent a late event during cervical carcinogenesis. In concordance with this, low fractions of cells harbouring PIK3CA mutations were found in invasive cervical carcinomas, indicating PIK3CA mutations are only present in subclones of the tumour [32], [38].

## 5 Conclusions

From the above summarised findings it becomes clear that following an infection with hrHPV, additional (epi)genetic alterations in host cell genes are crucial for the development of cervical (pre)cancerous lesions. The clinical implications of these alterations are thoroughly discussed by Steenbergen et al. [8]. The viral oncogenes directly or indirectly trigger the deregulation of many control mechanisms that ultimately lead to the accumulation of genetic and epigenetic alterations. Ultimately, the right combination of (epi)genetic alterations may provide a growth advantage and result in progression of a hgCIN to invasive cancer, a process that is estimated to take 15–30 years [39]. The key events may in part be different between cervical SCC and AdCA and dependent on the hrHPV type present.

**Fig. 1:**
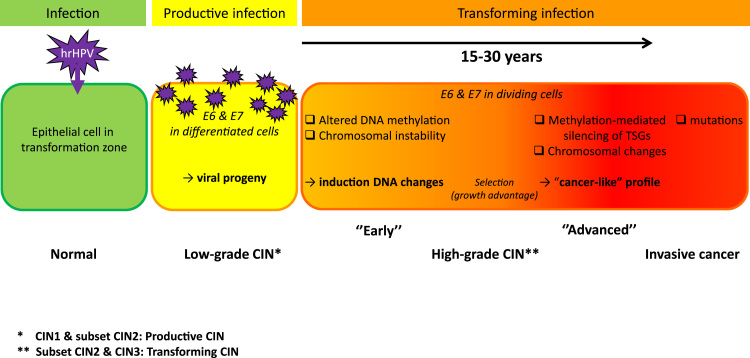
Schematic representation of HPV-mediated cervical carcinogenesis. Progression of a high-grade CIN lesion, characterized by viral oncogene expression in dividing cells (i.e. a transforming infection), to invasive cancer results from the accumulation of DNA changes induced by HPV. High-grade CIN represents a heterogeneous stage of disease with varying duration of existence (up to 30 years). ‘Advanced’ lesions show a cancer-like profile including hypermethylation of tumour suppressor genes and specific chromosomal alterations. Complementary somatic mutations only become detectable at the stage of invasive cancer. CIN: cervical intraepithelial neoplasia; TSG: tumour suppressor gene.

Based on the available information we propose a model (Fig. 1) in which a transforming infection by HPV contributes to deregulation of the DNA methylation machinery, which, upon selection may give rise to DNA methylation mediated silencing of tumour suppressor genes. Changes in overall DNA methylation as well as HPV-induced mitotic defects result in chromosomal instability and aneuploidy. Both increased DNA methylation of certain gene promoters and specific chromosomal alterations are primarily detectable in a subset of longer existing (i.e. advanced) hgCIN lesions that are likely to have a higher risk of progression to cancer. Mutations, on the other hand, are only very rarely found in hgCIN and in invasive cancers appear mostly restricted to specific subclones of cancer cells. Together, these observations indicate that mutations occur late in cervical carcinogenesis and are probably not among the most key molecular events involved in the progression of precancerous cervical lesions.

## Competing interests

RDMS has a minority stake in Self-Screen B.V., a spin-off company of VU University Medical Center Amsterdam.
